# 

**Published:** 1905-10-14

**Authors:** 


### NOTICE TO CORRESPONDENTS.

All MSS., Letters, Books for Review, and other matters intended for the Editor should be addressed The Editor, "The Hospital," The Hospital Buildings, 28 & 29 Southampton Street, Strand, W.C.

The Editor cannot undertake to return rejected MS., even when accompanied by stamped directed envelope.

**Notice to Advertisers and Subscribers.**

All Advertisements, Orders for Copies of The Hospital, and Business Communications should be addressed to **THE MANAGER (Not to the Editor),** The Hospital, 28 & 29 Southampton Street, Strand, London, W.C.

The Cost of Subscription, post free, is at follows:—Per week, 3½d.; per quarter, 3s. 3d.; per half-year, 6s. 6d.; per annum, 13s. Foreign and Colonial:—Per annum, 19s. 6d., payable, in all cases, in advance.

**NOTICE.—Vol. XXXVII. of "The Hospital" October 1904 to March 1905), in handsome cloth binding, will shortly be on sale at the office of this Journal, price 10s. 6d. Cases for binding the Numbers contained in this Volume 2s. Index to Vol. XXXVII., 3½d., post free.**

**The Monthly Part for October is now ready. Price 1s., by post, 1s. 3d.**

### BOOKS RECEIVED.

Baillière, Tindall, and Cox.

"Manual of Surgery." William Rose, M.B., B.S.Lond., F.R.C.S., and Albert Carless, M.S.Lond., F.R.C.S. 6th Ed.

"The Nature and Treatment of Cancer." By J. A. Shaw-Mackenzie, M.D.

J. and A. Churchill.

Apsley Cookery Book.

H. J. Glaisher.

"Indigestion." By G. Herschell, M.D. 3rd Ed.

Swan, Sonnenschein and Co.

"The Needs of Man." By Winslow Hall.

### Medical Appointments.

**Anderson, Thomas Lynewolde,** M.B., M.D.Melb., Medical Officer under the Workers Compensation Act, West Australia. **Blanchard, David Francis,** L.R.C.P. & S.Edin., L.F.P. & S.Glasg., Medical Officer under the Workers Compensation Act, West Australia. **Boyd, Alexander Brooke,** M.B., Ch.B.Oxon., Public Vaccinator for the District of Ashburton, New Zealand. **Brockbank, E. M.,** M.D., M.R.C.P.Lond., Honorary Assistant Physician to the Manchester Royal Infirmary. **Cornish, Charles Vivian,** M.R.C.S., L.R.C.P.Lond., Resident Medical Officer to the Hampstead General Hospital, N.W. **Eden, William Annesley,** L.R.C.P.Lond., M.R.C.S., L.S.A., re-appointed Medical Officer of the First District by the St. Columb (Cornwall) Board of Guardians. **Farmer, S.,** M.R.C.S.Eng., L.R.C.P.Edin., Certifying Surgeon under the Factory and Workshop Act for the Spennymoor District of the County of Durham. **Gallagher, J. O.,** M.D., C.M.Toronto, Clinical Assistant to the Chelsea Hospital for Women. **Griffin, J. V.,** L.R.C.P.I., L.R.C.S.I., Certifying Surgeon under the Factory and Workshop Act for the Crowborough District of the County of Sussex. **Haydon, Gerald Edgar Tom,** L.S.A., Officer of Health of Portland Shire, East Riding, Victoria, Australia. **Haynes, Edward James Ambrose,** L.R.C.P.Lond., F.R.C.S.Eng., Medical Officer under the Workers Compensation Act, West Australia. **Kinnear, J. A.,** M.D., C.M.Toronto, Clinical Assistant to the Chelsea Hospital for Women. **Lawes, C. H. E.,** M.B., Ch.M.Syd., Honorary Medical Officer to the Marrickville Cottage Hospital, New South Wales, Australia. **Leicester, J. C. Holdich,** M.D., B.S.Lond., F.R.C.S.Eng., Captain, I.M.S., First Surgeon to the Presidency General Hospital, Calcutta. **McAnally, Edward A.,** M.R.C.S.Eng., L.R.C.P.Lond., L.S.A., re-appointed Medical Officer of the No. 3 District of the Hollingbourn Union, Sittingbourne. **Mackenzie, John Hugh,** F.R.C.S.Edin., Acting Officer of Health of Flinders and Kangerong Shire, West Riding, Victoria, Australia. **McMahon, Frederick Dunbar Sutherland,** L.R.C.S., L.R.C.P., L.M.Edin., re-appointed Medical Officer for the Sixth District by the St. Columb Board of Guardians. **Mellish, John Stafford,** L.R.C.P.Lond., M.R.C.S., re-appointed Medical Officer for the Didmarton District by the Tetbury (Gloucestershire) Board of Guardians. **Miskin, Leonard John,** M.R.C.S., L.R.C.P., M.B., B.S.Lond., Medical Officer under the Workers Compensation Act, West Australia. **Owen, A. D.,** M.R.C.S., L.R.C.P.Lond., Medical Officer to Post Office, Hampton and Hampton Hill. **Williams, David Ernest,** L.M.R.C.P., L.M.R.C.S.Irel., Medical Officer under the Workers Compensation Act, West Australia.

### How to Become a Nurse

### THE NURSING PROFESSION:

**How and Where to Train.**

Edited by Sir HENRY BURDETT, K.C.B.

Gives full details of Training Schools, Premiums, Salaries, Hours of Duty, and all particulars required by anyone desirous of taking up Nursing as a profession.

### How to Succeed

It is essential to success that you should possess the best Text-books on Nursing and the Subjects pertaining thereto. We shall be pleased to assist you in this direction, and to supply any Works you may require.

*A COPY OF OUR CATALOGUE WILL INTEREST YOU.*

### A SELECTION—

**A HANDBOOK FOR NURSES.**
By J. K. WATSON, M.D., M.B., C.M.
New and Revised Edition. Profusely Illustrated.
5/- net, 5/4 post-free.

**A COMPLETE HANDBOOK of MIDWIFERY.**
**The Best Text-Book for the C.M.B.**
By J. K. WATSON, M.D.Edin.
143 Illustrations. 6/- net, 6/4 post-free.

**NURSING: ITS THEORY AND PRACTICE.**
**Being a Complete Text-book of Medical, Surgical, and Monthly Nursing.**
By PERCY G. LEWIS, M.D., M.R.C.S., L.S.A.
New Edition. Profusely Illustrated. 3/6 post-free.

**PRACTICAL GUIDE TO SURGICAL BANDAGING AND DRESSINGS.**
By WM. JOHNSON SMITH, F.R.C.S.
A handy and useful reference book. Suitable size for apron pocket.
2/- post-free.

**THE NURSES' PRONOUNCING DICTIONARY OF MEDICAL TERMS AND NURSING TREATMENT.**
By HONNOR MORTEN.
New Edition, with phonetic pronunciations.
Edited by MARY I. BURDETT. 2/- post-free.

**SURGICAL WARD-WORK AND NURSING.**
By ALEXANDER MILES, M.D.Edin., C.M., F.R.C.S.E.
Second Edition. With particulars and Illustrations of the Instruments used in various Operations. 3/6 net, 3/10 post-free.

**OPHTHALMIC NURSING.**
By SYDNEY STEPHENSON, M.B., F.R.C.S.E.
New and Revised Edition. 3/6 net, 3/10 post-free.

**ELEMENTARY ANATOMY AND SURGERY FOR NURSES.**
By WILLIAM McADAM ECCLES, M.S.Lond., M.B., F.R.C.S., L.R.C.P.
2/6 post-free.

**ELEMENTARY PHYSIOLOGY FOR NURSES.**
By C. F. MARSHALL, M.D., B.Sc., F.R.C.S.
A valuable help to Physiology for Probationers.
Crown 8vo, Illustrated, cloth. 2/- post-free.

**NURSING IN DISEASES OF THE THROAT, NOSE AND EAR.**
By P. MACLEOD YEARSLEY, F.R.C.S.Eng., M.R.C.S., L.R.C.P.Lond.
2/6 post-free.

**HOSPITAL SISTERS AND THEIR DUTIES.**

By EVA C. E. LÜCKES, Matron, London Hospital. Third Edition.

2/6 post-free.

**THE EXAMINATION OF THE URINE.**
**Compiled for the Use of Nurses.**
By J. K. WATSON, M.D., M.B., C.M. 1/- net, 1/1 post-free.

### SOME NEW BOOKS Ready Shortly.

**SURGICAL INSTRUMENTS AND APPLIANCES USED IN OPERATIONS.**
A Classified and Illustrated List, with Explanatory Notes.
By HAROLD BURROWS, F.R.C.S.
Limp cloth, 1/6 net, 1/8 post-free.
This work will prove of the utmost service as a work of reference to all Nurses.

**NURSING HINTS TO PROBATIONERS ON PRACTICAL WORK.**
By ANNESLEY VOYSEY.
Cloth, Illustrated, 2/- net, 2/4 post-free.
This work fills a distinct hiatus in nursing literature. It will prove of great assistance to the Nurse in the early stages of her career.

**MEDICAL ELECTRICITY AND THE LIGHT TREATMENT.**
By KATE NEALE,
Sister in Charge Light Department, Guy's Hospital.
Cloth, profusely Illustrated, 2/6 net, 2/9 post-free.
A practical book on a subject hitherto untouched from a nursing standpoint.

**THE NURSING OF SICK CHILDREN.**
By Dr. BURNET.
Cloth, 1/- net, 1/1 post-free.
A valuable little treatise on the nursing of children. It should prove useful to mothers as well as to Nurses.

**The Scientific Press, Ltd.**

*Complete Catalogue sent post free on application.*
Publishers of Nursing Text-Books, Charts, and Case-Books of all Descriptions.
28 & 29 SOUTHAMPTON STREET,
STRAND, LONDON, W.C.

